# Bristol Medico-Chirurgical Society

**Published:** 1973-07

**Authors:** 


### Bristol Medico-Chirurgical Society

The sixth meeting of the session was held at the Medical School on 14th March 1973. Dr. H. R. Cayton, Director, Public Health Laboratory Service, Bristol, addressed the Society on "A New Look at Some Old Fevers". Dr. Cayton also arranged a Bacteriological Demonstration and Dr. E. R. Davies showed skiagrams.

The following *Alterations to the Rules of the Society* were agreed:—

Rule 2 *add:*

Whenever the term 'member' is used without qualification in these rules it shall be deemed to include the classes of Honorary and Ordinary Members.

Rule 4 *add:*

An Honorary Member shall have the full privilege of membership of the Society but shall not pay an annual subscription.

Rule 7 *amend to:*

"A Meeting shall *normally* be held on the second Wednesday in each month, from October to May inclusive at the time arranged by the Committee *but the Committee shall have power to alter the date of any such Meeting.* The Meeting in October shall be the Annual Meeting, at which the election of Officers and Committee shall take place."

Rule 8 *amend to:*

8(a) The *Annual* subscription to the Society for an Ordinary member shall be £4.00 per annum *but this subscription will be reduced by a half* to £2.00 *per annum for those Ordinary members who have retired from their main appointment in the National Health Service.* The Annual subscription, which shall be payable in advance, *shall become due on the 1st day of October in any year.* This sum shall *entitle the member to attend and take part in any meeting of the Society, to vote at any such meeting,* to receive each issue of the Journal and to use the Library, with the privileges appertaining to the circulation of Library books.

8(b) *If the annual subscription due from any Ordinary member is unpaid by the 31st day of March, the Honorary Treasurer shall so inform the Committee at its next Meeting. Any Ordinary member whose annual subscription remains unpaid on the 30th day of September in any year may, by resolution of the Committee, be excluded from the Society and shall thereupon cease to be a member but such exclusion shall not relieve the member from the liability for the payment of the arrears of subscriptions due. By means of a letter sent to the member's address as known to to Society at least 14 days before the Committee next meets, the Honorary Treasurer shall inform a member whose subscription is unpaid on 31st day of March in any year that action is about to be taken under the terms of Rule 8(b).*

8(c) The Annual subscription to the Journal to non-members of the Society shall be £2.00 per annum payable in advance on the 1st day of January in any year.

Readers may be reminded that the Rules of the Society were printed in full in this Journal in October 1971 (Vol. **86**, p.77).

On 11th April on Open Meeting was held when Mr. George Twist, Q.P.M., Ll.M., Chief Constable of Bristol, spoke on "Police Work in a Changing Society". This was accompanied by a most interesting display of offensive weapons and other specimens of forensic interest.

The Final Meeting of the session on 9th May took the form of a Debate. The motion "That this House believes that the sales promotion activities of the Pharmaceutical Industry are contrary to the best interests of the National Health Service" was proposed by Dr. C. Hawkins, Consultant Physician, Birmingham and Dr. J. M. Naish, Consultant Physician, Bristol. It was opposed by Dr. B. W. Crombie, Chairman of Hoechst Pharmaceuticals and Mr. W. McMillan, Manager, Information Services, The Association of the British Pharmaceutical Industry. The Chairman was Dr. J. E. Cates. Dr. M. E. H. Halford arranged a pathological demonstration and Dr. F. G. M. Ross showed skiagrams.

At each of the above meetings Mr. B. P. Jones, Medical Librarian arranged a display of medical books.

